# Visualization Method for the Cell-Level Vesicle Transport Using Optical Flow and a Diverging Colormap

**DOI:** 10.3390/s21020522

**Published:** 2021-01-13

**Authors:** Seohyun Lee, Hyuno Kim, Hideo Higuchi, Masatoshi Ishikawa

**Affiliations:** 1 Information Technology Center, Data Science Research Division, The University of Tokyo, Hongo 7-3-1, Bunkyo-ku, Tokyo 113-8656, Japan; hyuno_kim@ds.itc.u-tokyo.ac.jp (H.K.); ishikawa@ishikawa-vision.org (M.I.); 2 Department of Physics, Graduate School of Science, The University of Tokyo, Hongo 7-3-1, Bunkyo-ku, Tokyo 113-0033, Japan; higuchi@phys.s.u-tokyo.ac.jp

**Keywords:** vesicle transport, optical flow, visualization

## Abstract

Elucidation of cell-level transport mediated by vesicles within a living cell provides key information regarding viral infection processes and also drug delivery mechanisms. Although the single-particle tracking method has enabled clear analysis of individual vesicle trajectories, information regarding the entire cell-level intracellular transport is hardly obtainable, due to the difficulty in collecting a large dataset with current methods. In this paper, we propose a visualization method of vesicle transport using optical flow, based on geometric cell center estimation and vector analysis, for measuring the trafficking directions. As a quantitative visualization method for determining the intracellular transport status, the proposed method is expected to be universally exploited in various biomedical cell image analyses.

## 1. Introduction

Vesicle refers to the package of intracellular nanoparticles that plays the role of a courier in a living cell [1]. Through constant interaction with the environment, cells engulf extracellular molecules and discharge unnecessary molecules based on the vesicle transports [2]. In particular, the uptake process, which is known as endocytosis, has been considered to include essential information for understanding the cell signaling and its regulation [3].

**Figure 1:**
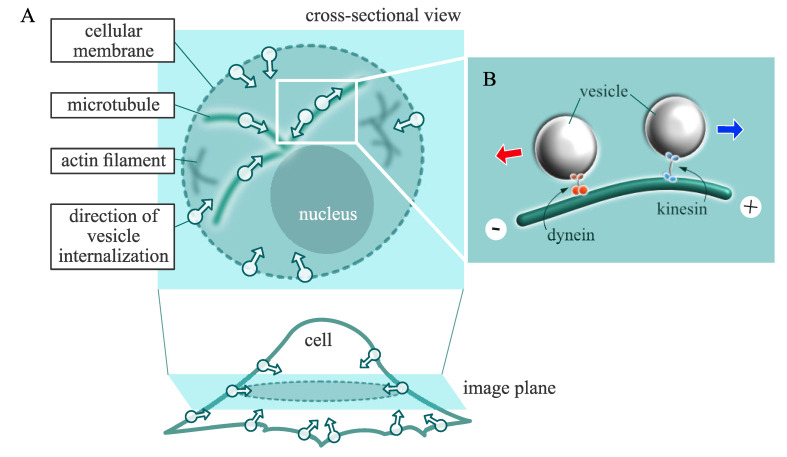
Concept of vesicle transport in a living cell. (**A**) Diagram of intracellular transport of quantum dot-labeled vesicle from cell cross-sectional view. Vesicles (White-colored circles with arrows) are internalized from the cellular membrane and then transported as they interact with cytoskeletal network, such as actin filament and microtubule. (**B**) Two different types of motor proteins, dynein and kinesin, for microtubule-based transport: Kinesins deliver vesicles in outward direction from the cell center, which is toward the plus end of the microtubule, while dyneins carry vesicles toward the microtubule minus end, which is approximately inward direction with respect to the cell center.

Since such information is closely related to elucidating the mechanism of intracellular communication between virus-infected cells and uninfected cells, signal cascades, and drug delivery [4,5], studies have been conducted to reveal the precise movement of vesicles, not only from the molecular analysis of motor proteins but also from the numerical analysis of the vesicle motion itself. Motor proteins indicate kinesin, dynein, and myosin, which directly carry the cargoes and mediate the interaction between vesicles and cytoskeletons [6]. The concept of vesicle internalization and transport by motor proteins in living cells is depicted in Figure 1. Majority of the studies on motor proteins have performed to measure their various physical properties, including stiffness and step size [7,8,9]. On the other hand, considerable analyses of three-dimensional vesicle trajectories using the single-particle tracking method have also been performed from the viewpoint of vesicle-cytoskeleton interaction [10,11,12,13].

Our understanding of the precise motion of vesicles in living cells has been greatly increased owing to the single-particle tracking method, as it detects the rotational motion of the vesicle along the cytoskeletal network [14,15,16] and categorizes the type of navigation between the cytoskeletons using machine learning approach [17]. However, though these studies have contributed to our current knowledge of single vesicle movement, the entire cell-level vesicle transport is not clearly understood yet.

The primary reason the cell-level image analysis for intracellular transport has not shown much progress is that the amount of vesicle trajectory data acquirable per cell using the single-particle method is limited. Analysis of a single vesicle trajectory does not offer enough information regarding the entire movement pattern of the vesicles at the whole cell level. Furthermore, fluctuation in the fluorescent dye intensity, which is inevitably caused by axial dynamics of the target vesicle, hinders us from finding a sufficient number of trackable cases. Therefore, it is inefficient to collect individual trajectories of vesicle using a single-particle tracking approach for understanding the whole cell-level intracellular transport.

One of the most promising solutions for the aforementioned problem is to apply computer vision techniques to cell image data analysis. In particular, optical flow, which is a computational method for detecting and estimating the pattern of the motions that appeared in a series of images, has been widely exploited in biomedical image analysis [18,19,20,21]. For the cell image analysis, optical flow has played a central role in the quantitative estimation of the embryonic cell aggregation and segregation [22], mitochondrial transport in neurons [23], the accumulation of specific proteins [24], cardiomyocyte contractile function [25], and the network sequence of cytoskeletal structures [26]. These applications have critically tested the performance and the applicability of the optical flow method for cell microscopy image data analysis.

In our previous study, we attempted to introduce the optical flow method for intracellular vesicle transport analysis, as a preliminary step to understand the whole cell-level vesicle trafficking pattern [27,28]. In the present study, we built a detailed model to apply the optical flow method combined with cell center estimation that can represent the direction of vesicle movement, implying motor protein involvement. Since the two types of major motor proteins, kinesin and dynein, move in opposite directions on microtubules, which spread from the central area of the cell, a diverging color map is suggested as a visualization method instead of conventional color space representation. In particular, we show the possibility of the intracellular transport analysis from the perspective of the distance from the cell center, by converting the cell image to a polar coordinate system.

## 2. Materials and Methods

### 2.1. Live Cell Imaging

The cell line used in the live cell imaging is KPL-4 human breast cancer cells, which was kindly provided by Dr. Kurebayashi [29] (Kawasaki Medical School, Kurashiki, Japan). The dish containing the cells was cultured in a complete growth medium (Dulbecco’s modified Eagle’s medium with high glucose, Nacalai Tesque, Inc., Kyoto, Japan) with 10% of fetal bovine serum (Thermo Fisher Scientific, Inc., Waltham, MA, USA), 1% of L-glutamine (Wako Pure Chemical Industries, Ltd., Osaka, Japan), and 1% of penicillin-streptomycin (Thermo Fisher Scientific, Inc., Waltham, MA, USA), and then stored in the incubator maintaining the temperature at 37 °C and CO_2_ level at 5%. To label the vesicles, 4 nM of carboxyl quantum dots (Qdot 655 ITK Carboxyl Quantum Dots, Thermo Fisher Scientific, Inc., Waltham, MA, USA) were added to induce endocytosis of cells for enhanced affinity to the cellular membrane [30,31]. Ten minutes after the incubation with the quantum dots, the cells were washed twice with PBS solution and heated with a heater (IN-ONI-F1, Tokai HIT, Shizuoka, Japan) installed on the microscope stage (IX70, Olympus, Tokyo, Japan), to maintain the physiological conditions.

**Figure 2:**
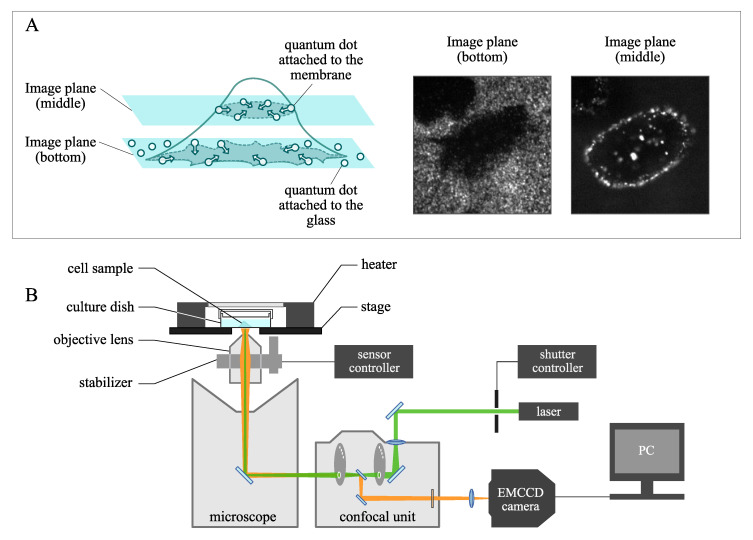
(**A**) Comparison between two cell images captured at the bottom and middle of the cell height. The imaging plane was empirically determined at 4 μm, considering the trade-off between signal-to-noise ratio and the intracellular area to be analyzed. (**B**) Structure of imaging system based on fluorescence confocal microscopy.

To capture the images near the middle of the cell, the axial position of the objective lens was adjusted and fixed with a capacitive sensor-based stage stabilizer, which controls the axial vibration within 4 nm in terms of standard deviation [32]. Cell images taken at the mid-height were preferred because internalization of the quantum dots is much more recognizable here compared with that at other cross-sections, such as those from the bottom or top of the cell. At the lowest section of the cell, since the quantum dots are likely to be attached not only to the cell membrane but also to the glass-bottom dish (Matsunami Glass Ind., Ltd., Osaka, Japan), the region of the cell is hardly recognizable. Additionally, if the images are taken at an excessively high axial position (as compared to images taken at the middle height of the cell), the cross section of the cell becomes smaller. In the experiment, considering the signal-to-noise ratio of the cell image with respect to the background, the position of the image plane from the bottom was empirically determined at 4 μm. A comparison between two cell images taken at the bottom and the middle of the cell height is illustrated in Figure 2A.

In the imaging system, quantum dot-labeled vesicles are excited by laser at 532 nm and emit the ray at approximately of 650 nm. The cross section of the target cell was taken with confocal microscopy for reduced signal-to-noise ratio, using a confocal scanner unit (CSU-10, Yokogawa Electric Company, Tokyo, Japan) and an EMCCD camera (Andor Ixon 885, Belfast, Northern Ireland) with an exposure time of 500 ms of for a total of 6 minutes at 1 Hz. Figure 2B shows the overall imaging system used in the live cell experiment.

### 2.2. Optical Flow Computation

After the cell images were acquired through time-lapse confocal imaging, the optical flows of the cell image data between adjacent frames were computed, based on the Lucas-Kanade method [33]. We chose the Lucas-Kanade method among various other schemes available for optical flow computation (such as Horn and Schunk method [34] and Farnebäck [35] scheme) because it is simple and efficient [19,36,37] and is proven suitable for biomedical image analysis, such as that of calcium signaling in a living cell [38].

The optical flow calculates the local displacement vectors between two subsequent image frames at each pixel. For fluorescence microscopy, which acquires localization information from the intensity distribution, optical flow can provide the direction and velocity information of the target. For an arbitrary pixel, *x*, *y*, with intensity I(x,y,t) in the image taken at time *t* which is displaced to I(x+δx,y+δy,t+δt) in two-dimensional image plane after δt, the optical flow vector V(x,y)=(δx/δt,δy/δt) satisfies Equation (1) based on brightness constancy premise [18,33].

(1) $$\nabla I\left(x,y,t\right)\;\cdot \;V\left(x,y\right)+\frac{\partial I(x,y,t)}{\partial t}=0$$

This is because the first order of Taylor expansion for the constraint, I(x,y,t)=I(x+δx,y+δy,t+δt), produces Equation (2).

(2) $$\frac{\partial I}{\partial x}V_{x}+\frac{\partial I}{\partial y}V_{y}+\frac{\partial I}{\partial t}=0$$

where $V_{x}$ and $V_{y}$ indicate the *x* and *y* components of V(x,y), respectively. Using this constraint, the Lucas-Kanade method computes the optical flow vector V(x,y) to minimize a weighted least-square fit of the constraint equation, after dividing the image into smaller section Ω, as shown in Equation (3).

(3) $$\sum_{x,y\in \Omega }W^{2}\left(x,y\right){\left[\nabla I\left(x,y,t\right)\;\cdot \;V\left(x,y\right)+\frac{\partial I(x,y,t)}{\partial t}\right]}^{2}$$

where *W* indicates the window function to apply more weight to the center in each section. Therefore, the optical flow vector V(x,y) can be computed using Equation (4).

(4) $$\left[\begin{array}{c} V_{x}\\ V_{y} \end{array}\right]={\left[\begin{array}{cc} \Sigma W^{2}I_{x}^{2} & \Sigma W^{2}I_{x}I_{y}\\ \Sigma W^{2}I_{y}I_{x} & \Sigma W^{2}I_{y}^{2} \end{array}\right]}^{-1}\left[\begin{array}{c} -\Sigma W^{2}I_{x}I_{t}\\ -\Sigma W^{2}I_{y}I_{t} \end{array}\right]$$

**Figure 3:**
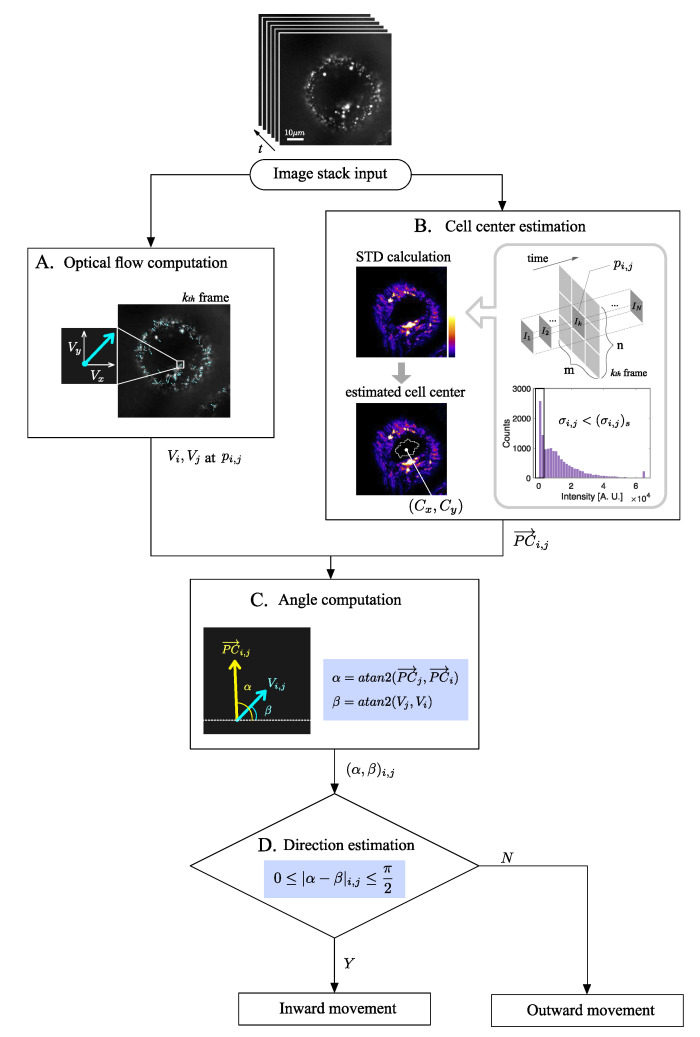
Workflow for estimating the direction of intracellular vesicle using optical flow and cell center estimation. (**A**) Optical flow computation based on Lucas-Kanade scheme [33] for the image stack. For simplicity of notation, let $V_{i},V_{j}$ represent the acquired optical flow at the pixel $p_{i,j}$. (**B**) Geometric cell center $\left(C_{x},C_{y}\right)$ as the mean coordinates of the area where intensity STD, $\sigma_{i,j}$, is smaller than one STD of the STD distribution, ${\left(\sigma_{i,j}\right)}_{s}$. ${\vec{PC}}_{i,j}$ is the vector from $p_{i,j}$ to $\left(C_{x},C_{y}\right)$. (**C**) Evaluation of angles α and β computed by four-quadrant inverse tangent of ${\vec{PC}}_{i,j}$ and $V_{i,j}$. ${\vec{PC}}_{i}$ indicates the *x*-component and ${\vec{PC}}_{j}$ refers to the *y*-component of the vector ${\vec{PC}}_{i,j}$, respectively. (**D**) Comparison of α and β to estimate the direction of vesicle with respect to the cell center.

In this study, the optical flow vectors V(x,y) for cell imaging data were calculated using the MATLAB function *estimateFlow* for the objects generated by the function *opticalFlowLK* which creates the optical flow objects based on the Lucas-Kanade method. The optical flow computation in the entire image processing procedure is as shown in Figure 3A.

### 2.3. Geometric Cell Center Estimation

To analyze the direction of optical flow with respect to the cell center, estimation of the geometric cell center is required. In this study, we exploited the distribution of intensity information of labeled vesicles inside the cell, as an example method to determine the cell center. Since the vesicles are internalized from the cellular membrane and gradually transported into the central area of the cell where the microtubule organizing center (MTOC) is located [39,40], the intensity fluctuation due to the movements of quantum dot-labeled vesicles can be considered to appear the least near the cell center, particularly in the early stage of endocytosis. On the other hand, the intensity fluctuation appears to be high in the periphery, including the membrane area. Therefore, time-series analysis of the imaging data is required to estimate the geometric center of the target cell.

For this purpose, the intensity standard deviation (STD) of the image stack can be used to estimate the cell center in the imaging data by comparing the STD values computed at each pixel. The standard deviation for a pixel $p_{i,j}$ of total N-frame cell image stack can be defined as $\sigma_{i,j}$ and calculated using Equation (5).

(5) $$\sigma_{i,j}=\sqrt{\frac{1}{N-1}\sum_{k=1}^{N}{\left(I_{k}\left(i,j\right)-\bar{I}\left(i,j\right)\right)}^{2}}$$

where $I_{k}\left(i,j\right)$ is the intensity of the pixel $p_{i,j}$ at *k*th frame and $\bar{I}\left(i,j\right)$ indicates the mean intensity value of $p_{i,j}$ for the entire N frames. In practice, the intensity standard deviation of each pixel for the entire imaging time, $\sigma_{i,j}$, was calculated using the image processing tools in *ImageJ* [41]. After the $\sigma_{i,j}$ for each pixel was acquired, the geometric center of the cell was estimated as the mean coordinates of the intracellular area, where the $\sigma_{i,j}$ is smaller than threshold value. The threshold value was determined again using the STD obtained from the distribution of $\sigma_{i,j}$. The threshold, $\sigma_{s}$, was defined as the STD of whole STD distribution and can be described as shown in Equation (6).

(6) $$\sigma_{s}=\sqrt{\frac{1}{mn-1}\sum_{i=1}^{m}\sum_{j=1}^{n}{\left(\sigma_{i,j}-{\bar{\sigma }}_{i,j}\right)}^{2}}$$

where *m* and *n* respectively refer to the total number of rows and columns of the image, and ${\bar{\sigma }}_{i,j}$ indicates the mean value of the STD distribution. To define the geometric cell center as $C_{x},C_{y}$, we introduce a weight function at each pixel $p_{i,j}$ as shown in Equation (7), $w_{i,j}$, for the sake of simplicity.

(7) $$w_{i,j}=\{\begin{array}{ccc} 1 & \mathrm{if} & \sigma_{i,j}<\left({\bar{\sigma }}_{i,j}-\sigma_{s}\right)\\ 0 & \mathrm{else} \end{array}$$

Then, $C_{x}$ and $C_{y}$ can be computed as mean coordinates of the area where $w_{i,j}=1$. With the definition of $\sum_{i=1}^{m}\sum_{i=j}^{n}w_{i,j}=\sum w_{i,j}$, the geometric cell center coordinate was calculated as shown in Equation (8).

(8) $$\left[\begin{array}{c} C_{x}\\ C_{y} \end{array}\right]=\left[\begin{array}{c} \frac{\sum (w_{i,j}\;\cdot \;i)}{\sum w_{i,j}}\\ \frac{\sum (w_{i,j}\;\cdot \;j)}{\sum w_{i,j}} \end{array}\right]$$

where *i* and *j* indicate the row and column of the pixel $p_{i,j}$ in the image, respectively. The processes of geometric cell center estimation for an image stack are shown in Figure 3B.

## 3. Result

### 3.1. Whole Cell Area Visualization Using Vector Comparison

As explained in the introduction section, intracellular transport of vesicles is mediated by motor proteins, dynein and kinesin, which work in opposite directions, for the majority of vesicles on the microtubules. Using the optical flow vector and the location of the geometric cell center, it is possible to visualize the vesicle movement at the cell level, estimating the time-series local distribution of motor proteins.

To do this, for each pixel $p_{i,j}$ in the image, a vector ${\vec{PC}}_{i,j}$, the direction of which indicates the cell center, was computed. Then, the angle between ${\vec{PC}}_{i,j}$ and the optical flow $V_{i,j}$ at $p_{i,j}$ was calculated using vector analysis. For simplicity, let the four-quadrant inverse tangent [42] of ${\vec{PC}}_{i,j}$ and $V_{i,j}$ be α and β, respectively. Because the α and β indicate the angles with respect to the *x*-axis by this computation, the degree of coherence for ${\vec{PC}}_{i,j}$ and $V_{i,j}$ can be defined by comparing α and β. The movement estimation at $p_{i,j}$ based on the comparison between α and β is as shown in Equation (9) and Figure 3C,D.

(9) $$\left\{\begin{array}{cc} \mathrm{Inward}\;\mathrm{direction}, & \mathrm{if}\;0\leq \left|\alpha -\beta \right|\leq \frac{\pi }{2}\\ \mathrm{Outward}\;\mathrm{direction}, & \mathrm{if}\;\frac{\pi }{2}<\left|\alpha -\beta \right|\leq \pi \end{array}\right.$$

Therefore, |α−β| can play a role as a parameter in estimating whether the optical flow at $p_{i,j}$ is moving toward the cell center or not, which is closely related to the type of motor protein involved in the vesicle movement. In particular, the above categorization is a good match with the diverging color map [43], which consists of two contrasting colors with tone gradient, for visualizing the intracellular transport at the cell level.

**Figure 4:**
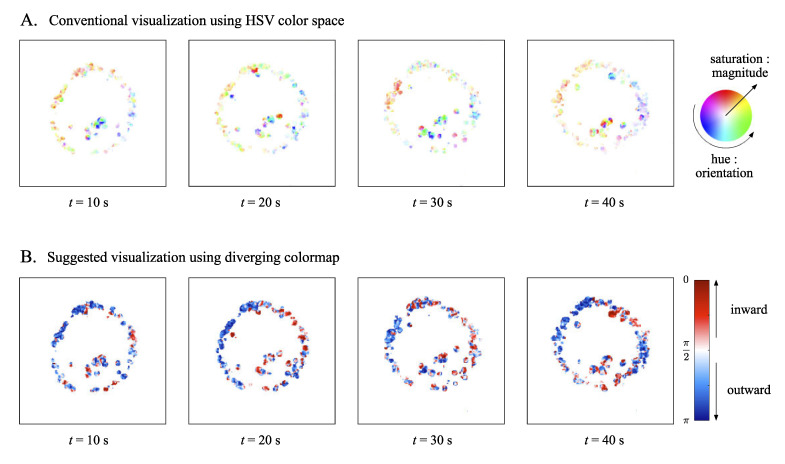
Comparison of visualization methods for vesicle movement in a living cell. (**A**) Conventional method using HSV (Hue, Saturation, Value) color space for optical flow. Hue indicates the orientation of optical flow vector, and saturation refers to the magnitude of the optical flow vector. (**B**) Suggested method using diverging colormap with two contrast colors. Blue color indicates the outward movement, while red color indicates inward movement with respect to the estimated cell center.

Figure 4 demonstrates the efficiency of the diverging colormap for visualizing the vesicle movement with respect to the cell center, compared with the conventional HSV color space expression. As shown in Figure 4A, the orientation of the optical flow vector at each pixel is colored with the corresponding hue, the saturation of which indicates the magnitude of the vector, in the HSV color space. With this, however, as the hue varies from 0° to 360° in two dimensions, biologically meaningful movement, which is closely related to the radial structure of intracellular transport, is hardly recognizable. In contrast, diverging colormap using |α−β| enables direct identification of the converging and diverging movement of the vesicle, with respect to the cell center.

### 3.2. Visualization of Endocytosis

**Figure 5:**
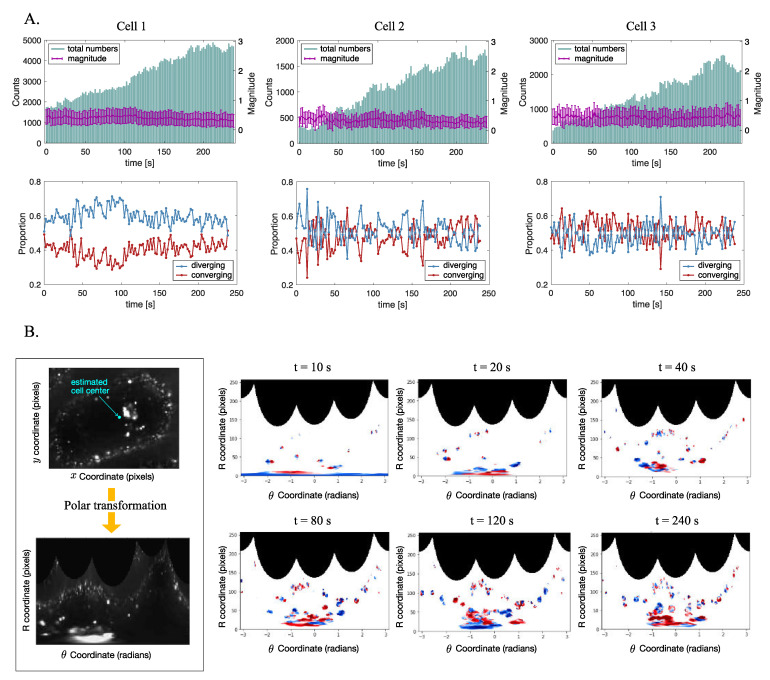
Direction of vesicle movement analysis using the suggested method. (**A**) Total numbers and magnitude of optical flow vectors detected over time for three sample cells (upper) and the proportion of diverging and converging movement over time (lower). (**B**) Polar transformation [44] of cell image to visualize the vesicle movement in terms of the distance from cell center (right). Vesicle movement of inward direction colored in red and outward in blue, after polar transformation is applied.

Using the process hitherto for acquiring the inward and outward movement of vesicle transport, endocytosis of living cells can be visualized from various perspectives. Figure 5 shows the examples of such applications. As shown in Figure 5A, in addition to the total numbers and magnitudes of the optical flow itself, the proportion of inward and outward movement of the vesicle with respect to the cell center can be obtained, as demonstrated with three sample cells.

Furthermore, by rearranging the cell area according to the distance from the center, it is possible to analyze which direction is more dominant at a specific time, which can be interpreted as the employment of motor proteins.

Polar transformation is one method can rearrange the cell image in terms of the distance from the cell center and the angle. The polar representation of the intracellular area can be performed using polar transformation of the image [44]. Based on the cell center, ($C_{x},C_{y}$), each pixel in the original Cartesian coordinates can be transformed into a polar image, as shown in Equation (10).

(10) $$\left(x,y\right)=\left(C_{x}+R\cos\theta ,C_{y}+R\sin\theta \right)$$

where *R* and θ indicate the distance and the angle between the cell center and the pixel (x,y), respectively. Therefore, in the polar representation, the original images with *x* and *y* coordinates in Cartesian coordinate system can be transformed into *R* and θ coordinates, which respectively indicate the distance and the angle with respect to the cell center. Figure 5B shows the process of polar transformation using fluorescent cell image (left) and the changes in vesicle transport direction in cell 1 as represented in the diverging colormap. In the converted polar coordinate system, users can easily recognize the feature of vesicle movement in terms of the distance from the cell center, which can be used in time-series image analysis-based cell experiments, such as virus infection test.

## 4. Discussion

Analyzing and understanding the movement of nanoscale particles, such as vesicle transport in living cells, has been a challenging topic in the field of biomedical image analysis. From individual vesicle tracking based on point spread function (PSF) analysis to recent integrated deep-learning approaches [45], various methods have been applied to explain the vesicle movement.

Although the tracking of each vesicle may have revealed the properties of individual vesicle transport, there remained shortcomings in complete understanding of the entire cell-level vesicle movement pattern. Optical flow is one of the promising computer vision techniques that can be applied to the vesicle transport imaging data analysis. Therefore, there have been many studies that exploited optical flow approach to understand cellular dynamics quantitatively: embryonic cell motion pattern analysis, velocity measurement for mitochondrial transport [22], to name a few [23]. However, optical flow approach in the previous studies has usually been used as a tool to calculate local dynamics in a cell, not from the view of the entire cell-level. In addition, visualization of optical flow with conventional HSV scale hardly relates to the intracellular structure, in that the optical flow vector represents the absolute angle in the image but not the biologically relevant angle in the cell.

Using the visualization method, in this study, we aimed to understand the pattern of vesicle transport at the whole cell level, based on the relative angle of optical flow and the estimated position of the cell center. Because the suggested method combines the optical flow approach with the cell center estimation algorithm, the computed optical flows now can represent the biologically meaningful direction, as determining whether or not the vesicle is moving toward cell center, which is the direction of endocytosis. Additionally, compared to the conventional visualization of optical flow using the HSV space, this new method enables visualization of the direction of the vesicle with respect to the cell center using two contrast colors, which can be interpreted as the involvement of motor proteins for the vesicle transport. Since the detailed features of two major types of motor proteins, dynein and kinesin which carry cargoes in the opposite directions along microtubules, have not yet been clearly understood in the whole cell-level, visualization method using contrasting colormap is expected to significantly help future analysis on the involvement status and the dynamics of the motor proteins in living cells.

**Figure 6:**
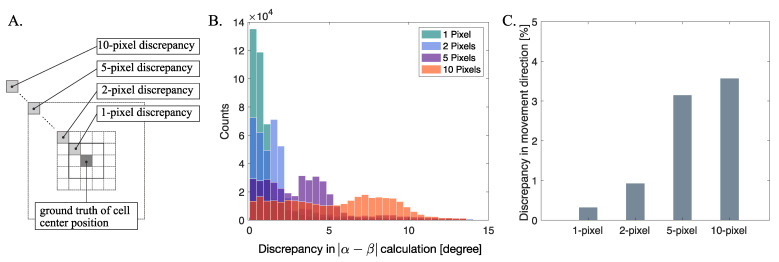
Influence of cell center estimation error in applying the suggested method. (**A**) Concept of discrepancy in cell center estimation for 1, 2, 5, and 10 pixels. (**B**) Representative result of computing the error sizes of |α−β| for a single cell experiment. (**C**) Discrepancy in movement direction represented in percentage compared to the ground truth, according to the discrepancy in cell center estimation in pixels.

While we estimated the geometric center of the cell as the mean position of the intracellular area where the standard deviation of the intensity fluctuation appears the least, imaging MTOC with installing additional optical path may improve the accuracy of cell center detection. Although the MTOC was not imaged in this study so as not to reduce the imaging intensity in confocal microscopy, direct localization of MTOC can contribute to a more accurate calculation of directions of the vesicles. The range of errors in the direction angles according to the possible error size of the cell center localization is demonstrated in Figure 6. Because the larger discrepancy of cell center localization in pixels causes a larger error range in the |α−β| angle calculation, as we tested with 1, 2, 5, and 10 pixels, accurate localization of the cell center is one of the important factors for practical applications of the suggested method.

Along with the accuracy issue regarding the cell center position, approximate parameters for optical flow computation and noise level of the image should be considered before applying the suggested method. Because these issues depend upon the specific imaging conditions of each user, and many studies have already dealt with related methods [18,19,20,21], discussing the details of such parameters is beyond the scope of this work.

One of the concerns of the users who consider applying the suggested method to their system might be regarding the image region of the cell that can be subjected to the analysis. Although we basically recommend users to use individual cells that are separated, image processing method for detecting active contour such as Snakes [46] is expected to help find the cellular area of a specific target cell. In addition, because the proposed method assumes the vesicles moves within the depth of the image plane, three-dimensional movements of some vesicles that can be shown as appearing or disappearing in the image plane might be wrongly detected. In this case, multi-plane imaging [47] is expected to help exclude such spurious detection, by comparing the images acquired from vertically separated nearest image planes.

One of the major advantages of adopting the suggested methods is that various cell experiments conducted to examine the features of biological processes including endocytosis can be analyzed visually. In particular, virus infection, drug delivery, cell communication, signal cascade, and inhibition tests for a living cell, which require time-series whole cell-level analysis, can benefit from the visualization method proposed in this study. For example, in the inhibition experiment, users can quantitatively analyze the overall intracellular transport from the membrane to the nucleus periphery by measuring the direction changes using different reagents and juxtaposing the control experiment results. In particular, the proposed method can accelerate our understanding of fundamental cellular processes that occur with the bidirectional or radial property. Not only the elongation of the fibrils from the initial seed [48] but also the radial capillary flows which is also known as coffee ring effect [49] can be visualized with the suggested approach to understand the precise kinetics of the protein self-assembly, measuring the overall dynamics in the growth of the target structures. Furthermore, we expect that the presented method can be applied to quantification of the cytoplasmic structure, by supplementing the readout of the biosensor [50] regarding the position inside the cell, if the process is automated and computationally optimized for video-rate tracking [51], which remains as our future work.

## 5. Conclusions

In this paper, we have presented an endocytosis visualization method to examine the vesicle movement toward the cell center. Based on the conventional optical flow of the cell image, the location of the cell center was estimated using intensity changes. By comparing the angle between the optical flow vector and the cell center-oriented vector, each movement was analyzed to represent the relational direction, exploiting the diverging color map. Because the two major motor proteins, dynein and kinesin, which deliver intracellular cargoes, move in opposite directions on microtubules organized in the cell center area, the proposed method is expected to significantly help understand intracellular transport under various scenarios, including virus infection process and drug delivery.
